# Optimization of urban green space in Wuhan based on machine learning algorithm from the perspective of healthy city

**DOI:** 10.3389/fpubh.2025.1490857

**Published:** 2025-03-06

**Authors:** Xuechun Zhou, Xiaofei Zou, Wenzuixiong Xiong

**Affiliations:** ^1^School of Social and Political Sciences, University of Glasgow, Glasgow, United Kingdom; ^2^School of Art, Hubei University, Wuhan, China

**Keywords:** machine learning, random Forest algorithm, Support Vector Machine algorithm, optimization of urban green space, healthy city

## Abstract

**Introduction:**

Urban green spaces play a critical role in addressing health issues, ecological challenges, and uneven resource distribution in cities. This study focuses on Wuhan, where low green coverage rates and imbalanced green space allocation pose significant challenges. Adopting a healthy city development perspective, the research aims to assess the impact of green space optimization on urban health, economic performance, and social structure.

**Methods:**

A multivariable model was constructed using random forest and Support Vector Machine (SVM) algorithms to evaluate the influence of key indicators on urban green space. Core indicators were integrated from three dimensions: residents' health, environmental quality, and community interaction. Multiple linear regression analysis was employed to quantify the potential benefits of green space optimization on economic and social outcomes.

**Results:**

The findings reveal that optimizing health and environmental quality indices significantly enhances green space development. Green space improvements drive a 73% increase in economic efficiency by improving residents' health and extending life expectancy. Additionally, enhancements in social structure are achieved at rates of 61% and 52% through strengthened community cohesion and improved environmental quality, respectively. The model demonstrates high stability and adaptability after multiple iterations, providing a robust quantitative foundation for green space optimization.

**Discussion:**

This study highlights the multidimensional value of green space optimization in promoting urban health, economic growth, and social stability. The results offer a solid theoretical basis and practical guidance for green space planning and management in healthy cities, contributing to scientific decision-making and sustainable urban development.

## 1 Introduction

Amid rapid technological advancements, the accelerated urbanization process has led to a sharp increase in population density and resource allocation pressures, making urban green space planning and management an urgent issue (1, 2). As an important central city in China, Wuhan faces uneven distribution of green space resources in its urban areas, with an overall insufficient green area and a need for further improvement in green coverage. This situation poses significant challenges to both residents' quality of life and the sustainability of the urban ecosystem (3–5). The lack of urban green space may not only lead to a continuous decline in environmental quality but also significantly exacerbate residents' psychological stress and physical health issues.

Therefore, a comprehensive investigation into the intricate relationship between the optimization of green space layout and residents' health is crucial for understanding the mechanisms through which urban green spaces influence living environments. Such research can provide essential theoretical and practical support for enhancing urban planning and improving residents' quality of life (6).

In recent years, research exploring the relationship between urban green space and residents' health has advanced significantly. Studies have increasingly employed machine learning (ML) and spatial modeling techniques to examine the role of green space in optimizing living environments. For instance, Wu (7) developed a livability prediction model for Dutch cities using ML algorithms. By integrating features through decision jungles and decision forests, the model achieved over 90% prediction accuracy. This study highlighted that air pollution was a key factor affecting urban livability and demonstrated that green space, acting as an ecological buffer, could mitigate the effects of air pollution. ML techniques have proven to be highly effective in processing dynamic data and updating knowledge, offering valuable insights for analyzing complex urban ecosystems. Furthermore, Tella et al. (8) demonstrated the efficacy of the Random Forest (RF) algorithm in air pollution modeling, accurately predicting PM10 hotspots in Selangor, Malaysia. They revealed that the spatiotemporal distribution of air pollution could reflect deficiencies in green space in urbanized areas, which could exacerbate the negative health impacts of pollution exposure. While these studies have emphasized the environmental buffering role of green space, they have given less attention to its direct health benefits for urban populations.

From the perspective of urban expansion and land use, Elhamdouni et al. (9) analyzed the dynamic expansion of Khenifra city in Morocco from 1991 to 2017 using SVM techniques. Their findings revealed that the urban spatial occupancy rate surged from 12% to 36%, accompanied by a notable imbalance in the expansion pattern. This highlighted the potential adverse effects of rapid urbanization on the equity and spatial balance of green space distribution, which in turn exacerbates the reduction of residents' activity spaces and weakens community cohesion. In a similar vein, Chowdhury (10) compared the classification performance of ML algorithms and identified the high efficiency of SVM, RF, and Artificial Neural Networks (ANNs) in land use and cover classification, providing a scientific approach to modeling urban spatial dynamics. While these studies offer valuable technical insights into land use and urban expansion, they have not fully explored how the lack of green spaces indirectly affects overall quality of life by impacting residents' mental health and social interactions. As such, further systematic research is required to explore the specific roles of green spaces in enhancing both urban ecological and social functions.

The key findings of existing research can be summarized in two main points: first, the optimization of urban ecosystem stability and environmental quality through green space; and second, the positive impact of green space on residents' mental health and community interactions. However, from a critical perspective, there are still some issues in the current research. Most studies remain at the level of single-factor analysis and lack comprehensive research that considers urban environments, social factors, and other dimensions. Building upon these considerations, this study, based on machine learning algorithms, integrates both the RF and Support Vector Machine (SVM) algorithms to construct a new multivariable model. This model aims to systematically reveal the complex relationship between the layout of green space and residents' health in Wuhan's urban areas. Through nonlinear fitting and feature weight analysis, the model precisely captures the interactions between green space and various health indicators. Additionally, by optimizing kernel parameters and selecting decision tree features, it enhances the explanatory power of the relationship between health city indicators and green space, enabling data-driven prediction and optimization. Ultimately, the model provides a scientific basis for achieving health and sustainable urban development.

## 2 Relevant ideas of urban green space optimization based on ML from the perspective of healthy city

### 2.1 Healthy city

According to the World Health Organization (WHO) definition in 1994, a healthy city is one that places human health at its core, ensuring a healthy living and working environment for its citizens through systematic efforts in urban planning, construction, and management. The goal is to achieve the organic integration of healthy populations, environments, and societies (11). In recent years, this concept has been widely adopted, with its core focus on promoting comprehensive urban health development through improvements in environmental quality, the health of residents, and community cohesion (12, 13). The development of a healthy city requires the coordination of multiple sectors, including environmental protection, public health services, the implementation of social and economic policies, as well as community development and resident participation (14). For example, increasing urban green spaces and public open areas not only enhances residents' physical and mental health but also promotes community cohesion and social interaction (15). Studies have shown that the development of healthy cities can improve residents' quality of life, foster social harmony, and promote sustainable economic growth (16).

This study extracts key indicators from extensive research and practice, categorizing them into three main domains: residents' physical health, urban environmental quality, and community cohesion and interaction. First, urban green spaces are closely linked to residents' physical and mental health. Contact with natural environments has been shown to reduce psychological stress, improve mental health, and increase the frequency of physical activities (17). Second, optimizing urban environmental quality has a direct impact on improving residents' health. Properly planned green spaces can reduce air and noise pollution, thereby enhancing the overall environmental quality (18). Finally, community cohesion plays a vital role in healthy cities. The provision of green spaces and public areas not only strengthens community interaction and a sense of belonging but also promotes the integration and shared development of diverse cultures (19). These factors are interwoven, collectively forming a comprehensive system for healthy city development.

### 2.2 Optimization of urban green space

The optimization of urban green space involves enhancing both the quantity and quality of green areas through scientifically informed planning and management. This process aims to improve the urban ecological environment and, consequently, the quality of life for residents. Urban green spaces not only offer aesthetic value and recreational opportunities but also play a crucial role in climate regulation, air and soil purification, and water conservation (20). For example, urban green spaces can significantly mitigate the urban heat island effect by providing shade and evaporative cooling, thus lowering local temperatures. Additionally, these spaces contribute to improving air quality by absorbing carbon dioxide and other pollutants (21). Optimizing the layout of urban green spaces can bolster the stability and sustainability of urban ecosystems, while also enhancing residents' wellbeing and overall health (22, 23). Research has demonstrated that green spaces can substantially reduce the urban heat island effect, adjust local temperatures, and promote evaporative cooling (24, 25). Furthermore, green spaces improve air quality by capturing carbon dioxide and other pollutants, which in turn reduces the incidence of respiratory illnesses (26, 27). Properly optimizing the distribution of urban green spaces contributes to ecosystem stability and sustainability while simultaneously improving residents' health and happiness. Studies have shown that residents living in proximity to green spaces report better mental health and more frequent social interactions compared to those in areas without access to green space (28, 29). Additionally, the rational allocation of green spaces, along with efforts to increase their accessibility and diversity, enables better fulfillment of the needs of various age groups and social sectors. This promotes social equity and enhances public health and wellbeing (30). Modern urban planning should embrace a multi-center approach, creating self-sustaining urban communities that reduce residents' dependency on automobile transport while improving overall health and safety (21). This model has been successfully implemented in various global cities, such as the “15-min city” concept in Paris, where residents can meet all their daily needs within a 15-min walk or bike ride (31). Through these strategies, optimizing urban green spaces not only enhances environmental quality but also provides residents with improved health and social opportunities, thereby fostering the sustainable development of the city as a whole.

### 2.3 The ML algorithm

Machine learning (ML), as a critical branch of artificial intelligence, enables computers to learn from data and continuously optimize their performance by analyzing data, recognizing patterns, and building predictive models (32). Based on statistical and mathematical principles, ML constructs mathematical models to identify patterns in data, thereby automatically improving the performance of computational systems (33). RF and SVM are two classic machine learning algorithms that are widely applied due to their excellent performance in classification and regression tasks. The differences between these two algorithms are not only reflected in their specific implementation mechanisms but also in the types of scenarios they are best suited for. RF is particularly suitable for datasets with large amounts of redundant features and noise, as it can efficiently extract key features and perform classification tasks. On the other hand, SVM excels in solving nonlinear problems due to its ability to accurately describe complex decision boundaries and optimize classification margins. The two algorithms demonstrate highly complementary characteristics when addressing multidimensional and complex issues. RF offers stability and generalization ability, while SVM focuses on optimizing classification performance for nonlinear data. This complementary advantage provides a scientifically sound and efficient solution for handling complex datasets.

**Figure 1:**
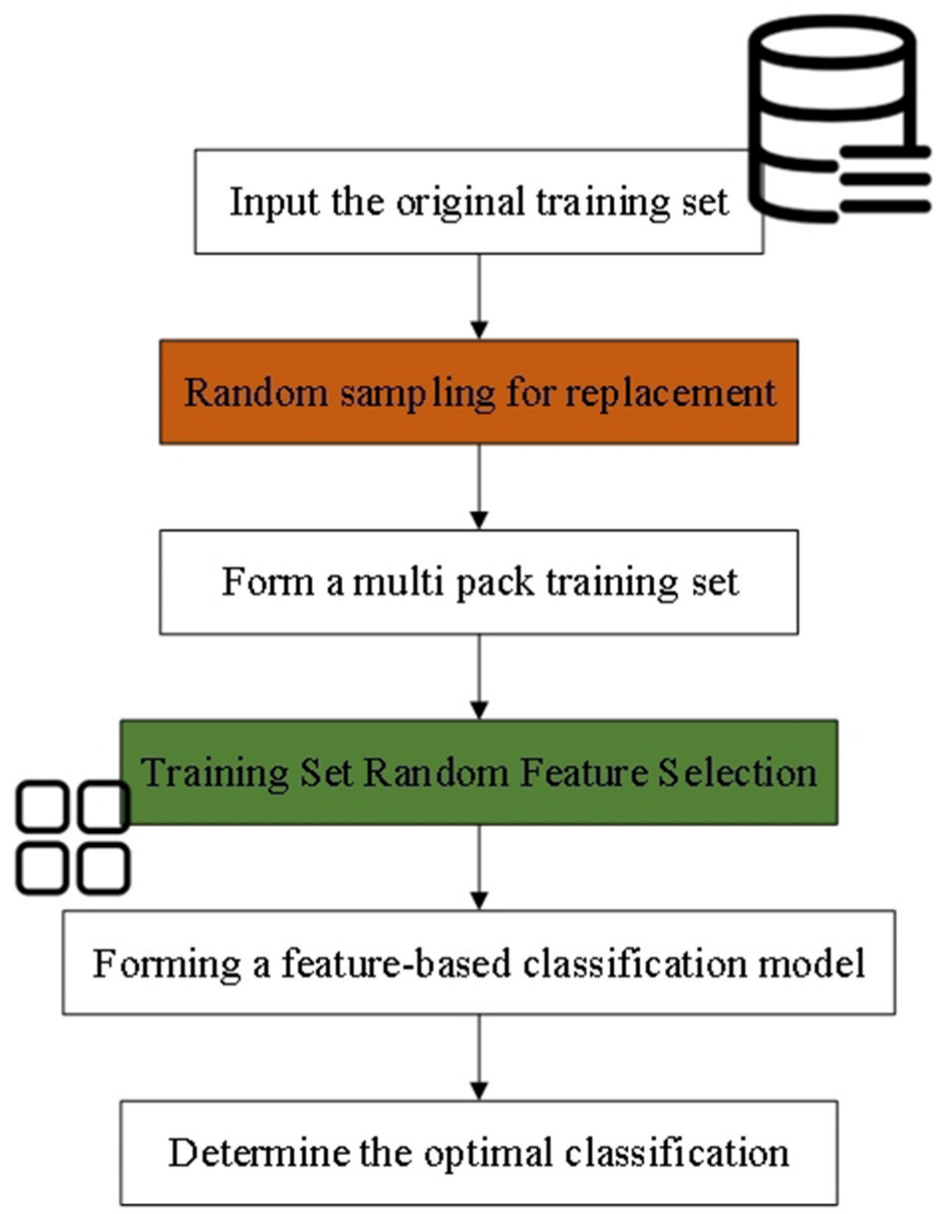
The implementation process of RF.

RF is an ensemble learning method that performs classification and regression tasks by constructing multiple decision trees. The core concept of RF lies in ensemble learning, where the fundamental unit is the decision tree. In essence, multiple weak classifiers (decision trees) are combined to create a strong classifier, thereby enhancing the overall performance of the model (34, 35). The implementation process of RF is illustrated in Figure 1.

**Figure 2:**
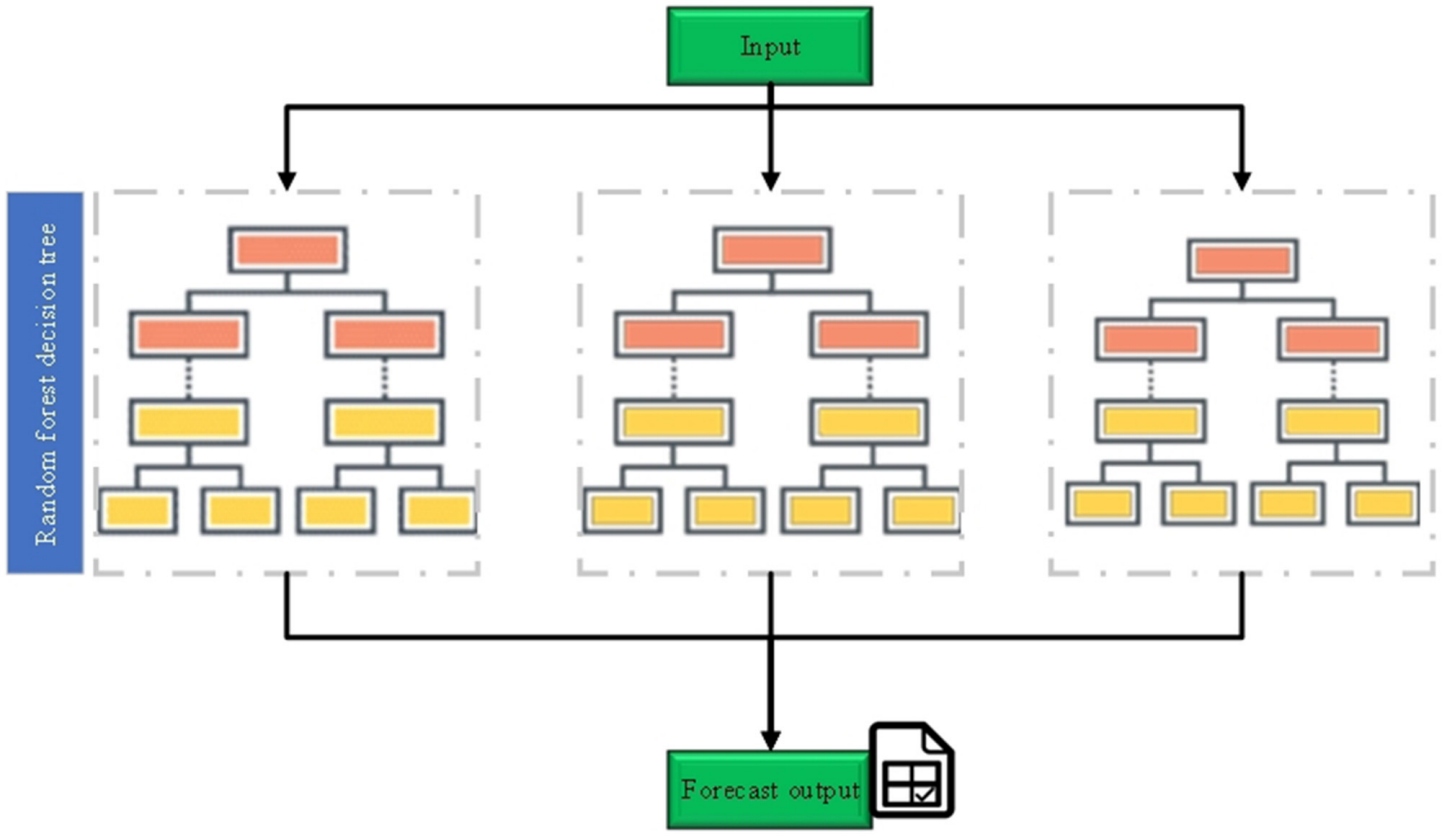
Schematic diagram of RF implementation.

In RF, each decision tree is constructed using randomly selected features and samples. This randomness reduces the model's variance and enhances its generalization ability (36). When constructing each decision tree, RF ensures diversity by employing bootstrap sampling and random feature selection, which helps differentiate each tree within the ensemble (37, 38). The operational principle of RF is illustrated in Figure 2

SVM is a powerful supervised learning method designed for classification and regression tasks (39, 40). The core idea of SVM is to maximize the margin between distinct sample points by identifying an optimal hyperplane (41). In classification tasks, SVM maximizes the distance between the nearest support vector and the decision boundary (42). When the data is not linearly separable, SVM employs a kernel function to map the data to a higher-dimensional space, where an optimal hyperplane can be identified in the nonlinear space (43).

**Figure 3:**
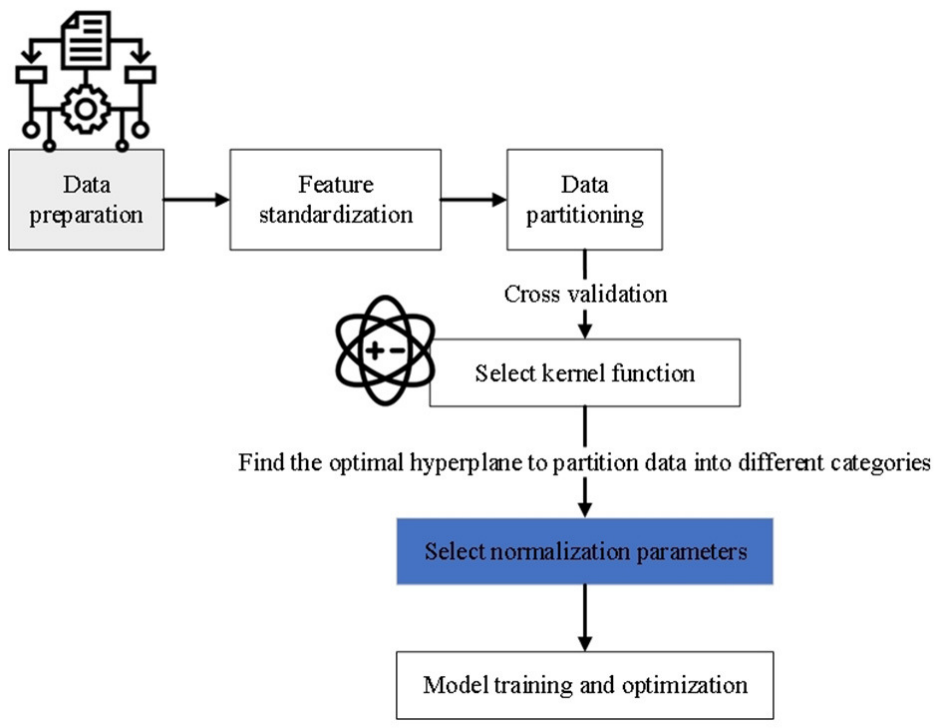
Flow chart of SVM implementation.

The advantage of SVM lies in its strong generalization ability and its adaptability to high-dimensional data. It is particularly effective when dealing with small sample sets and nonlinear data. The core principle of SVM is to identify the optimal classification boundary by maximizing the margin between classes, thereby improving the model's robustness and classification accuracy (44). The flowchart depicting the implementation process of SVM is shown in Figure 3.

## 3 Multivariable model design of Wuhan urban green space based on the ML algorithm

Urban green spaces play a vital role in addressing climate change. They serve as natural infrastructure for regulating microclimates and mitigating the urban heat island effect. Additionally, they help reduce carbon emissions and enhance urban ecological resilience. Extreme climate events triggered by climate change—such as heatwaves, droughts, and heavy rainfall—have profoundly affected the stability and functionality of green space systems. Specifically, rising temperatures intensify evaporation and reduce soil moisture content, significantly impacting the growth cycles and biodiversity of vegetation within green spaces. Changes in precipitation patterns cause uneven spatial and temporal distribution of rainfall. This increases the complexity of water resource management in green spaces. As a result, their ecological functions and residents' quality of life are affected. Moreover, extreme weather events such as heavy storms and heatwaves are becoming more frequent. These events raise management costs and pose challenges to green space infrastructure, plant growth, and ecosystem service functions.

From an ecosystem services perspective, the relationship between urban green spaces and climate change is characterized by multiple interactions. Urban green spaces can effectively mitigate the negative impacts of climate change by expanding vegetation coverage, enhancing soil water retention, improving air quality, and regulating temperature and humidity. As the “carbon sinks” of cities, green spaces absorb and store carbon dioxide, thereby playing a crucial role in slowing climate change. However, climate change itself imposes new challenges on green space systems. High temperatures and drought put stress on plants in green spaces. Some may experience growth stagnation, reduced adaptability, or even death. This weakens their ability to sequester carbon and provide ecological protection. Additionally, extreme climate events such as heavy rainfall and flooding are becoming more frequent. These events cause soil erosion and waste water resources in green spaces. They may also lead to the collapse of ecological structures, further increasing urban vulnerability to climate change.

This study employs a multivariable modeling approach to explore the relationship between urban green spaces and healthy cities. It integrates diverse data on climate, the environment, and public health to reveal these complex interactions. The analysis of green spaces in urban Wuhan incorporates multiple indicators, including residents' health, environmental quality, and community interactions, to construct a comprehensive research framework. To capture the nonlinear relationships among these multidimensional data, this study utilizes two powerful machine learning algorithms: RF and SVM. These algorithms were selected due to their superior performance in handling complex datasets, uncovering latent patterns, and optimizing predictive accuracy.

In terms of theoretical methodology, the RF algorithm offers high tolerance and adaptability, while SVM excels in solving nonlinear problems in high-dimensional spaces. Specifically, RF learns from data by constructing multiple decision trees, employing bootstrap sampling and random feature selection during tree generation. This enhances the model's adaptability to complex relationships while reducing the risk of overfitting due to data noise. Furthermore, RF's ensemble nature allows it to process diverse input features, making it well-suited for heterogeneous urban health and environmental data, ensuring more accurate and stable predictions. Meanwhile, SVM achieves data classification by identifying the optimal decision boundary that maximizes the margin between support vectors and the hyperplane. When handling nonlinear relationships, SVM employs kernel functions to map data from lower-dimensional to higher-dimensional spaces, enabling the construction of more precise classification models. Notably, by optimizing kernel function parameters through grid search and cross-validation, the model's classification capability in complex data environments can be further enhanced. This advantage makes SVM particularly effective in analyzing the multifaceted impacts of urban green spaces on healthy city indicators, as it can identify subtle and latent associations.

The index data used in this study are sourced from publicly available official datasets, including annual statistical reports, environmental quality monitoring reports, and health survey data published by the Wuhan Municipal Bureau of Statistics and the Environmental Protection Bureau. As these datasets are publicly accessible, ethical concerns are not involved. However, potential sample selection biases may arise. For example, some indices may not fully represent the spatial distribution of Wuhan due to sampling being concentrated in specific urban districts. Additionally, certain environmental quality data may be limited by fixed monitoring schedules, which may hinder the capture of dynamic trends. To mitigate the impact of these biases, all raw data undergo a preprocessing stage, during which data cleaning is performed to address missing values and outliers. For missing data imputation, the k-nearest neighbors (k-NN) method based on similar samples is employed. This approach fills in missing values by calculating the mean of the nearest neighbors in the feature space, effectively preserving the overall relationships within the data. Specifically, for each missing value, the Euclidean distance between the target sample and other samples is first computed. The sample values of the k-nearest neighbors are then selected as references and imputed according to a weighted similarity, thereby retaining the intrinsic structural information of the dataset. For outlier detection, the 3σ rule is applied. The mean and standard deviation of each variable are calculated, and any values outside the range defined by the mean ± three times the standard deviation are identified as outliers and removed. This step prevents extreme values from distorting the model analysis. Subsequently, the Min-Max normalization method is used to standardize the data across different dimensions, scaling all values to the [0, 1] range to ensure consistency and comparability. To further optimize the dataset, Principal Component Analysis (PCA) is employed to reduce dimensionality, minimizing redundant information and enhancing the efficiency and accuracy of the model. During the PCA process, the number of principal components is selected based on a cumulative variance contribution rate of 95%. This dimensionality reduction not only improves data quality and consistency but also provides a solid foundation for the subsequent machine learning models. After preprocessing, the dataset is transformed such that each sample contains a set of standardized features, effectively reflecting the relationship between urban green space and healthy city indices in Wuhan.

This study adopts a multi-dimensional and multi-level approach to ensure a comprehensive and scientifically rigorous analysis. The selection of indices is designed to accurately reflect the real-world conditions and functional characteristics of urban green space. In line with established research literature and guidelines on urban greening and the development of healthy cities, the chosen indices are both scientifically sound and practically applicable. Specifically, fundamental indices such as green area, vegetation coverage rate, and green coverage density directly measure the extent and distribution of urban green space. These indices are commonly employed in urban ecology and environmental science research. Additional indices, such as the length of green corridors and the number of parks, offer further insights into the function and structure of green space, emphasizing its connectivity and accessibility for residents. These factors are significant in urban planning and design. Moreover, the garden quantity index captures the actual use and perception of green space by residents, making it a critical indicator for evaluating the social benefits and community interactions facilitated by these spaces. The indices used in this study for Wuhan are summarized in Table 1.

**Table 1:** Indices of Wuhan.

**Type**	**Index**	**Content**
Indices of urban green space in Wuhan	Green area	The total area covered by green space in Wuhan includes parks, green belts, greenways, and other green spaces.
	Vegetation coverage	The proportion of vegetation coverage in the urban area of Wuhan reflects the degree of vegetation greening in the city.
	Green coverage density	It measures the distribution density of green space such as green space and green belt in Wuhan, that is, the number of green coverage per unit area.
	Green corridor length	The total length of all kinds of green corridors in the urban area of Wuhan, including tree-lined roads and riverside green belts, is an important channel connecting urban green spaces.
	Number of parks	The total number of parks in the urban area of Wuhan reflects the distribution of leisure and entertainment space in the city.
	Green coverage rate	The proportion of green space (including green space, green belt, etc.) in the urban area of Wuhan is an important index to evaluate the degree of urban greening.
	Number of gardens	The number of gardens within courtyards, communities, and public areas in Wuhan, such as community and school gardens, provides greenery and venues for resident activities.
Health indices of Wuhan residents	Health condition	The overall health status of Wuhan residents, including physical and mental health.
	Chronic disease incidence	The chronic disease incidence among Wuhan residents, including hypertension, diabetes, cardiovascular and cerebrovascular diseases, etc.
	Average life span	The average life expectancy of Wuhan residents reflects the overall health level and quality of life.
	Health consciousness	Wuhan residents' awareness and concern about health problems, including the mastery of health knowledge and health care awareness.
Urban environmental quality index of Wuhan	Air quality index	It reflects the concentration levels of various pollutants in the air of Wuhan, including PM2.5, PM10, sulfur dioxide, nitrogen dioxide, etc. It has an important impact on residents' health and environmental quality.
	Noise level	It measures the intensity and frequency of environmental noise in Wuhan, encompassing traffic, industrial, and community noise. It impacts residents' quality of life and physical and mental health.
	Water quality index	It is critical to reflect the concentration and water quality of various pollutants in Wuhan water. It includes heavy metals, organic pollutants, microorganisms, and so on, for residents' domestic water use and ecological environment protection.
	Soil pollution index	It measures the concentration and distribution of various pollutants in the soil of Wuhan, including heavy metals, organic pollutants, and pesticide residues. It has an important impact on the safety of agricultural products and the protection of the ecological environment.
	Light pollution index	It reflects the night light intensity and light pollution degree in Wuhan, covering the number and intensity of urban night lighting facilities. It has an impact on the quality of life of residents and the biological ecological environment at night.
Community interaction index of Wuhan	Frequency of community activities	It reflects the frequency and participation of various community activities in the Wuhan community, encompassing cultural and sports activities, voluntary services, social gatherings, etc.
	Community organization density	It measures the number and distribution density of various community organizations in the Wuhan community, including community neighborhood committees, industry committees, and volunteer organizations.
	Community interaction participation index	It reflects the communication and interaction among community residents in Wuhan, involving neighborhood relations, participation in community activities, community information transmission, etc.
	Utilization of community public facilities	It shows the utilization degree of various public facilities in the Wuhan community, encompassing fitness facilities, libraries, cultural activity centers, etc. It reflects the activity degree of the community and the utilization efficiency of public resources.

The selection of these indices aligns closely with the study objectives. First, indices such as green area and vegetation coverage directly reflect the scale and quality of urban green space. These factors are crucial for improving residents' health, enhancing air quality, and regulating urban microclimates. Second, indices such as green coverage density and green corridor length provide insights into the distribution and connectivity of green space, facilitating the creation of a cohesive green grid and enhancing the overall functionality of the urban ecosystem. The number of parks and gardens serves as a measure of green space accessibility and utilization, which directly influences residents' outdoor activity levels and the extent of community interaction. By examining these indices, the impact of green space on residents' health, urban environmental quality, and community vitality can be thoroughly evaluated, providing a solid scientific foundation for optimizing green space layout. Ultimately, this approach supports the goal of building a healthy and livable city.

In this study, the parameters of the ML algorithms are optimized to ensure the model's high accuracy and robustness. The objective of parameter optimization is to enhance the model's adaptability and predictive performance by adjusting key parameters in the algorithm, thereby achieving the study goal of optimizing green space in Wuhan. The parameter optimization process is informed by both the characteristics of the algorithms and the specific nature of the data. Specifically, the two primary ML methods used—RF and SVM—each require the optimization of unique parameters. For RF, the focus is on parameters such as n_estimators, max_features, max_depth, and min_samples_split, which directly influence the model's complexity and predictive accuracy. The optimal combination of these parameters is identified through a systematic adjustment process. For SVM, key parameters including the kernel function, regularization parameter (C), gamma, and epsilon value play a critical role in determining the SVM's ability to map and classify data in high-dimensional space. The parameter optimization process for SVM involves grid search and cross-validation, allowing for the selection of the most suitable combination of parameters. The parameter optimization details for both RF and SVM are summarized in Table 2.

**Table 2:** Optimization process of ML algorithms.

**Algorithm**	**Parameter**	**Initial value**	**Optimization process**	**Optimal parameter value**
RF	n_estimators	100	Start from 100, gradually increase, and select the number that maximizes the model effect.	300
	max_features	Automatic	Trying different feature numbers [including sqrt(n_features) and log2(n_features)], and selecting the best feature number.	sqrt
	max_depth	None	Start with None and increase gradually until the model effect is no longer improved.	20
	min_samples_split	5	Step by step, select the number of segmentation samples that can maximize the model effect.	2
SVM	kernel	Radial Basis Function (RBF)	Trying different kernel functions (including RBF, linear kernel and polynomial kernel) and choosing the kernel function that is most suitable for the data.	RBF
	regularization parameter(C)	1	Through grid search or cross-validation, the optimal C value is selected from a certain range.	1
	gamma	scale	According to the different kernel functions, the optimal gamma value is selected.	scale
	epsilon	0.01	Choosing the optimal epsilon value from a certain range makes the tolerance of the model to errors more reasonable in different situations.	0.1

In the design of the multivariate model, RF performs random sampling and combines numerous features through feature selection and predictive model construction. This approach effectively identifies key features that significantly influence the dependent variables, thereby enhancing the prediction accuracy and stability of the model (45, 46). The primary mathematical process involved is as follows:

- Decision Tree Segmentation Calculation

To implement RF, it is necessary to determine the influence of each feature on the target variable through decision tree segmentation. This is achieved by calculating the Information Gain (IG), which quantifies the improvement in information provided by a feature when dividing the data. The feature with the highest IG is selected to split the data, thereby constructing the decision tree nodes. The specific calculation is represented in Equation 1:


(1) $$\begin{array}{l} Gain\left(X,Y\right)=Entropy\left(X\right)-\overset{n}{\underset{i=1}{\sum }}\frac{\left|X_{i}\right|}{\left|X\right|}\times Entropy\left(X_{i}\right) \end{array}$$

*X* refers to a feature set. *Y* represents the target variable. |*X*_*i*_| stands for the size of the *i-th* subset in feature *X*. |*X*| denotes the total size of feature set *X*. *Entropy*(*X*) indicates the entropy of feature set *X*. In this way, the optimal features can be selected for segmentation to maximize the IG of the data.

- Information entropy calculation of decision tree nodes

The information entropy calculation at decision tree nodes measures the uncertainty inherent in the data. Higher information entropy indicates greater uncertainty, while a lower entropy suggests less uncertainty. During the construction of a decision tree, features that most effectively reduce information entropy are selected, thereby minimizing data uncertainty and enhancing the predictive power of the tree. The calculation of information entropy is given by:


(2) $$\begin{array}{l} Entropy\left(X\right)=-\overset{m}{\underset{i=1}{\sum }}p_{i}\times \log_{2}\left(p_{i}\right) \end{array}$$

*p*_*i*_ represents the proportion of class *i* on the node. By calculating the information entropy, the effect of data segmentation with different features can be evaluated, and the features that can minimize the uncertainty can be selected for segmentation.

- Output of decision tree leaf nodes

Once the decision tree is constructed, each leaf node generates a predicted value, which is obtained by averaging the target variable values of all samples within the node. This approach effectively utilizes all available information in the leaf node to produce stable prediction outcomes. The output of the decision tree leaf node is calculated as follows:


(3) $$\begin{array}{l} {\hat{y}}_{tree}=\frac{1}{N}\overset{N}{\underset{i=1}{\sum }}y_{i} \end{array}$$

*N* represents the sample number of leaf nodes. *y*_*i*_ represents the target variable value of sample *i*. Through this averaging method, the final predicted value of each leaf node can be obtained.

- Output of RF

Building on this, the final prediction output of the RF is derived by aggregating the predicted values from multiple decision trees. Specifically, the RF output is the average of the predictions from all trees, which serves to reduce the prediction error inherent in individual decision trees and enhances the stability and accuracy of the overall model. The RF output is represented by the following equation:


(4) $$\begin{array}{l} {\hat{y}}_{forest}=\frac{1}{T}\overset{T}{\underset{t=1}{\sum }}{\hat{y}}_{tree_{t}} \end{array}$$

*T* represents the number of trees in the *RF*. ŷ_*tree*_*t*__ refers to the predicted output of the *t* tree. Through this calculation, RF can synthesize the prediction results of multiple decision trees and provide a more stable and accurate prediction.

- Calculation of feature importance

In this study, RF determines the features that most influence the model's prediction outcomes by assessing the importance of each feature. This process is carried out by aggregating the IG across all decision trees. Calculating feature importance provides insights into which variables contribute most to the model's predictive power. The calculation is expressed as follows:


(5) $$\begin{array}{l} Importance\left(X_{i}\right)=\overset{T}{\underset{t=1}{\sum }}Gain\left(X_{i},Y_{t}\right) \end{array}$$

*X*_*i*_ represents the *i*-th feature. *Y*_*t*_ refers to the target variable of the *t-th* tree. The features that have a significant impact on the prediction results can be identified by calculating the importance of features, thus optimizing the model's performance.

- Error calculation

Finally, the model's prediction accuracy and goodness of fit are evaluated through error analysis. Typically, the performance of the RF model is measured by computing the mean squared error (MSE) between the predicted and actual values. The error calculation is given by the following equation:


(6) $$\begin{array}{l} Error_{forest}=\frac{1}{T}\overset{T}{\underset{t=1}{\sum }}{\left({\hat{y}}_{tree_{t}}-y\right)}^{2} \end{array}$$

*y* represents the true value of the target variable. Error calculation is used to assess the model's prediction accuracy and goodness of fit. Based on the aforementioned algorithm and calculation process, RF is capable of capturing complex nonlinear relationships in large-scale, multidimensional data, thereby enhancing the model's robustness and generalization ability. As a result, RF provides a solid scientific foundation for optimizing urban green space in Wuhan.

SVM operates by mapping the data into a high-dimensional feature space to identify a hyperplane that maximizes the margin between different categories, thereby facilitating effective classification and prediction. In the design of a multivariable model, SVM is particularly useful for uncovering nonlinear relationships in the data, enabling effective classification and regression. This improves the model's ability to express and fit complex data relationships. The core mathematical process is outlined as follows:

## 4 Calculation of the decision function in a linear SVM classifier

SVM begins by determining the classification boundary through the calculation of the linear decision function. This decision function maps the input samples into a high-dimensional space, where classification is based on the distance between the samples and the decision hyperplane. The equation for the decision function of a linear SVM is as follows:


(7) $$\begin{array}{l} \hat{y}\left(x\right)=w^{T}\cdot x+b \end{array}$$

ŷ(*x*) represents the prediction category of the input sample *x*; *w* and *b* refer to model parameters. Through calculation, SVM can classify the input data into different categories.

- Calculation of objective function of linear SVM classifier

In linear SVM, the objective function is used to optimize the classification hyperplane by maximizing the margin between support vectors while minimizing classification errors. This objective function incorporates both the classification margin and an error term, which together enhance the model's classification performance. The equation is expressed as:


(8) $$\begin{array}{l} \underset{w,b}{\min}\frac{1}{2}∥w{∥}^{2}+C\overset{n}{\underset{i=1}{\sum }}\max\left(0,1-y_{i}\left(w^{T}\cdot x_{i}+b\right)\right) \end{array}$$

*x*_*i*_ and *y*_*i*_ respectively represent training samples and their corresponding categories. *C* stands for regularization parameter. Thus, SVM can find an optimal hyperplane that balances the classification interval and classification error.

- Calculation of decision function (kernel skill) of nonlinear SVM classifier

For data that is not linearly separable, SVM utilizes a kernel function to map the data into a higher-dimensional space, where it identifies the optimal hyperplane that maximizes the margin. The use of kernel methods enables the handling of complex data structures, thereby achieving more accurate classifications. The decision function equation for nonlinear SVM is:


(9) $$\begin{array}{l} \hat{y}\left(x\right)=\overset{n_{sv}}{\underset{i=1}{\sum }}\alpha_{i}y_{i}K\left(x,x_{i}\right)+b \end{array}$$

*n*_*sv*_ represents the number of support vectors. α_*i*_ represents the Lagrange multiplier of support vector. *K*(*x, x*_*i*_) represents the kernel function. Through kernel function, SVM can classify in high-dimensional space and deal with nonlinear problems.

- The objective function calculation of SVM under kernel technique

In the kernel method, the objective function of SVM is employed to optimize the margin between support vectors, thereby enhancing classification performance by maximizing this margin. The equation integrates the Lagrange multiplier of the support vectors and the kernel function, enabling effective classification of nonlinear data. The calculation is expressed as:


(10) $$\begin{array}{l} \underset{\alpha }{\min}\frac{1}{2}\overset{n_{sv}}{\underset{i=1}{\sum }}\overset{n_{sv}}{\underset{j=1}{\sum }}\alpha_{i}\alpha_{j}y_{i}y_{j}K\left(x_{i},x_{j}\right)-\overset{n_{sv}}{\underset{i=1}{\sum }}\alpha_{i} \end{array}$$

The objective function calculation of SVM based on the kernel technique can find the optimal hyperplane in high-dimensional space and realize the accurate classification of complex data.

- The Karush-Kuhn-Tucker (KKT) condition of the dual problem is as follows:

During the SVM optimization process, the KKT conditions are applied to ensure the optimality of the solution. The KKT conditions are crucial for solving optimization problems, as they guarantee that the solution is globally optimal. The equation representing the KKT conditions is as follows:


(11) $$\begin{array}{l} \{\begin{array}{c} \alpha_{i}\geq 0\\ y_{i}\left(w^{T}\cdot x_{i}+b\right)-1\geq 0\\ \alpha_{i}\left[y_{i}\left(w^{T}\cdot x_{i}+b\right)-1\right]=0 \end{array} \end{array}$$

By satisfying the aforementioned conditions, SVM effectively captures nonlinear relationships in complex, multidimensional data, ensuring the optimal solution to the optimization problem and enabling accurate classification and prediction.

In this study, the Analytic Hierarchy Process (AHP) is employed to determine the weight of each index within the composite index. First, a hierarchical structure model is constructed, categorizing the indices into target, criterion, and index layers. The target layer aims at optimizing urban green space, while the criterion layer includes indices related to health, environmental quality, and community interaction. The index layer further refines these into specific quantitative metrics. This hierarchical approach ensures a systematic and structured determination of weights. In the operational process, professional judgment and evaluation are used to conduct pairwise comparisons of the indices at each level, determining their relative importance. A pairwise comparison matrix is then constructed, where each matrix element represents the relative importance ratio between two indices. The weights are calculated using the feature vector method, and the comparison matrix is normalized. The initial weight for each index is derived by calculating the maximum eigenvalue of the matrix and its corresponding eigenvector. To ensure consistency within the comparison matrix, the Consistency Ratio (CR) is calculated. The CR is determined by the ratio of the consistency index to the random consistency index, as outlined in Equation 12:


(12) $$\begin{array}{l} CR=\frac{CI}{RI}=\frac{\lambda_{\max}-n}{\left(n-1\right)\times RI} \end{array}$$

λ_max_ is the largest eigenvalue of the comparison matrix. *n* means the order of the matrix. *RI* refers to the random consistency index, and *CI* denotes the consistency index. If the CR value is less than 0.1, it is considered that the matrix has good consistency. Otherwise, it is necessary to re-evaluate and adjust the element values in the comparison matrix.

In this study, three feedback optimization iterations were conducted. After the first optimization, the CR value was 0.15, exceeding the acceptable threshold of 0.1. Consequently, the results were returned to the expert group, with specific areas for improvement identified, prompting a re-evaluation and re-scoring. Following the second optimization, the CR value was reduced to 0.11. Although there was some improvement, the value still failed to meet the consistency requirements, necessitating further feedback, evaluation, and adjustment. After the third optimization, the CR value was successfully reduced to 0.08, meeting the consistency standard of <0.1. This ensured the consistency of the comparison matrix. Through these three optimization iterations, the final weights for each index were determined, accurately reflecting their relative importance in the comprehensive evaluation. The weights of each dependent variable index, categorized by type, are presented in Table 3.

**Table 3:** Weight of each dependent variable index under each type.

**Index**	**Health indices of Wuhan residents**	**Urban environmental quality index of Wuhan**	**Wuhan community interaction index**
Green area	0.25	0.15	0.12
Vegetation coverage	0.20	0.18	0.10
Green coverage density	0.15	0.12	0.08
Green corridor length	0.10	0.20	0.05
Number of parks	0.08	0.14	0.20
Green coverage rate	0.07	0.10	0.22
Number of gardens	0.05	0.11	0.23

To comprehensively assess the optimization of urban green space in Wuhan, this study introduces the calculation of a comprehensive index, which encapsulates the collective impact of several key factors on green space. This index reflects the interrelationships and significance of environmental quality, health, and community interaction indices. After determining the weight of each index through the AHP, these weights are applied to the actual data, enabling the quantification and holistic evaluation of each factor's contribution to urban green space. The calculation of the comprehensive index is expressed in Equation 13:


(13) $$\begin{array}{l} SI=\frac{\sum_{i=1}^{n}w_{i}*\left(p_{i}+\frac{1}{q_{i}}\right)}{\sum_{j=1}^{m}\sum_{k=1}^{l}\left(\frac{x_{jk}}{\sum_{k=1}^{l}x_{jk}}\right)*\left(1+\frac{y_{jk}}{\sum_{k=1}^{l}y_{jk}}\right)} \end{array}$$

Equation 13 integrates the scores and quantized values of the independent variables and urban green space indices. It computes the comprehensive index by taking the weighted average of the respective variables, alongside the normalized weighted sum of the urban green space indices. The equation's complexity arises from the combination of polynomial and fractional operations, reflecting the intricate relationships among multiple factors in the comprehensive evaluation. Here, *w*_*i*_ represents the weight of each variable index, *p*_*i*_ and *q*_*i*_ denote the score and quantized value of the independent variable, respectively. *y*_*jk*_ indicates the quantitative value of the urban green space index, while *n* and *l* correspond to the number and classification of the independent variable indices. *x*_*jk*_ and *m* refer to the score and count of urban green space indices.

## 5 Verification and analysis of multivariable models using ML algorithms

### 5.1 Regression analysis and verification of Wuhan residents' health

**Figure 4:**
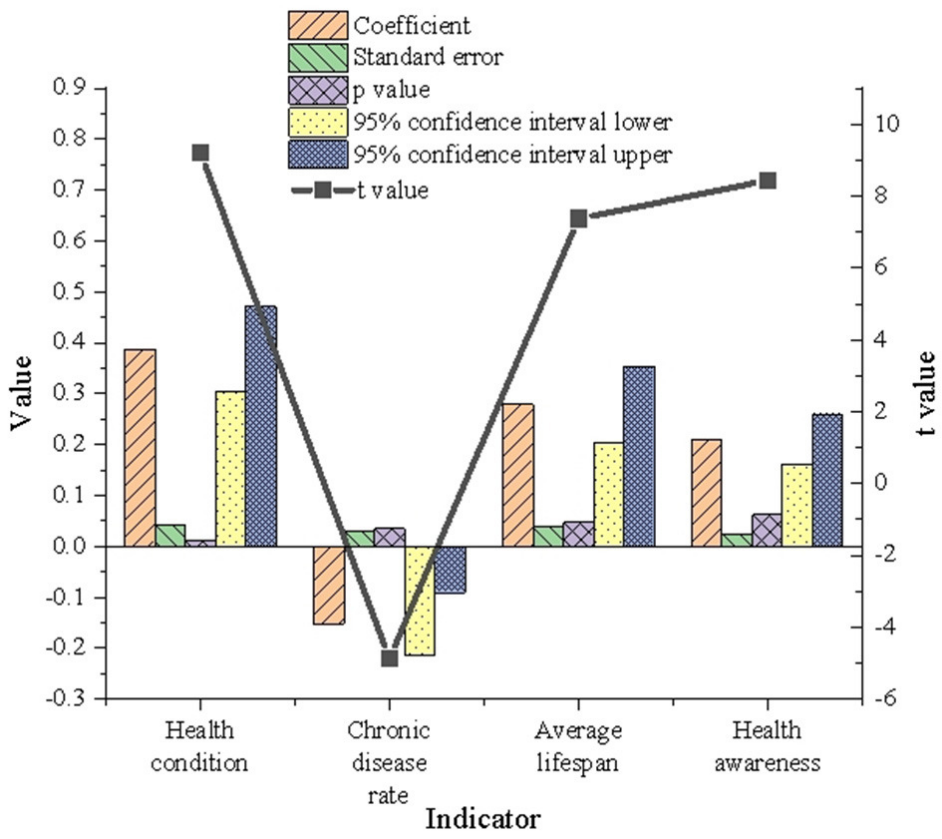
Verification results of multivariate model on residents' health in Wuhan.

The multivariate model's verification and analysis results for Wuhan residents' health are revealed in Figure 4.

Figure 4 illustrates the regression results for the health condition index, with a coefficient of 0.387, a *t*-value of 9.214, and a *p*-value of 0.012. These values indicate that the health condition index significantly and positively influences urban green space. Similarly, the regression coefficient for average life expectancy is 0.279, with a *p*-value of 0.047 (*p* < 0.05), further demonstrating its significant positive effect on urban green space development. Conversely, while chronic disease incidence is statistically significant, its regression coefficient is −0.152, with a *p*-value of 0.035 (*p* < 0.05). This suggests a negative relationship between chronic disease incidence and urban green space. The impact of health awareness, although characterized by a regression coefficient of 0.211, appears less substantial, as its *p*-value exceeds the threshold for statistical significance. These findings underscore that enhancing residents' health levels and increasing average life expectancy contribute positively to the development and optimization of urban green spaces. Consequently, improvements in urban green infrastructure have the potential to elevate overall environmental quality and the quality of life for residents.

The regression analysis also highlights the critical role of urban green spaces in health promotion, particularly in enhancing life expectancy and overall health levels. However, the negative association observed with chronic disease incidence reveals potential regional disparities in the health benefits of green spaces, which may be attributed to underlying socioeconomic factors. Areas with high chronic disease prevalence are often characterized by limited green space availability and inadequate infrastructure. Residents in such areas, exposed to prolonged environmental stressors, may exhibit diminished health benefits from green spaces and an increased dependency on medical resources. Additionally, individuals with chronic diseases may use green spaces less frequently, further limiting the health-promoting effects of these areas. The findings highlight the need for strategic optimization of green space layouts to address disparities in accessibility and equity, particularly for vulnerable populations. Efforts to increase green coverage in densely populated areas, enhance infrastructure, and incorporate health-oriented design principles can significantly amplify the positive health impacts of green spaces. Such measures are essential for fostering the holistic development of healthy and sustainable urban environments.

### 5.2 Regression analysis and verification of urban environmental quality in Wuhan

**Figure 5:**
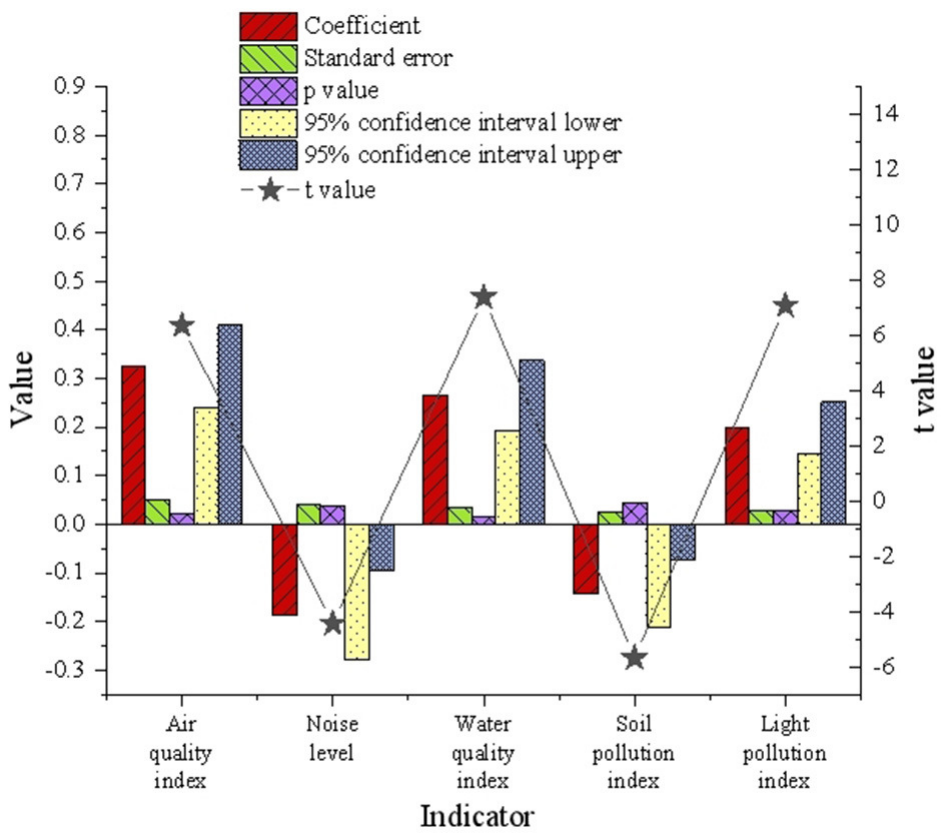
Verification results of multivariate model on urban environmental quality in Wuhan.

The multivariate model's verification and analysis results for urban environmental quality in Wuhan are illustrated in Figure 5.

Figure 5 illustrates the degree and direction of the impact of Wuhan's urban environmental quality indices on urban green space. The regression analysis reveals positive coefficients for air quality (0.324), water quality (0.265), and light pollution (0.198), indicating a positive correlation between these indices and urban green space. This suggests that improved environmental quality is associated with an increase in urban green space. Conversely, the regression coefficients for noise levels (−0.187) and soil pollution (−0.143) demonstrate a negative relationship with urban green space, signifying that deteriorating environmental conditions adversely affect green space availability. The *t*-values for noise levels (−4.429) and soil pollution (−5.684) further emphasize the significant influence of these factors on urban green space. The reliability of these results is supported by the 95% confidence interval's upper and lower bounds. These findings highlight the importance of enhancing air quality, water quality, and minimizing light pollution to support urban green space development. Simultaneously, addressing noise pollution and mitigating soil contamination are critical for improving overall environmental quality and fostering the expansion and optimization of urban green spaces.

A further analysis of these findings underscores not only the value of green spaces in improving environmental quality but also highlights their role in mitigating the urban heat island effect from a climate change perspective. Specifically, vegetation transpiration can reduce local urban temperatures, alleviating the adverse health impacts of extreme heat events on residents. Additionally, the soil and water retention functions of green spaces help alleviate the pressure on urban water systems during extreme rainfall events, demonstrating their critical regulatory role in the context of climate-induced extreme weather. Moreover, increasing ecological corridors and vegetation coverage in high-pollution areas can effectively reduce noise diffusion and the risks of soil contamination while enhancing the capacity for ecosystem services. From the intersectional perspective of climate change and healthy urban development, these measures provide actionable pathways for improving environmental quality and practical evidence for enhancing urban resilience.

Therefore, optimizing green space layouts should prioritize reducing noise and soil pollution while improving air and water quality indices. Through the planning of green buffer zones and the implementation of soil remediation projects, green spaces can simultaneously enhance environmental quality and ecosystem services, contributing greater value to the sustainable development of cities in the context of climate change.

### 5.3 Regression analysis and verification of community interaction in Wuhan

**Figure 6:**
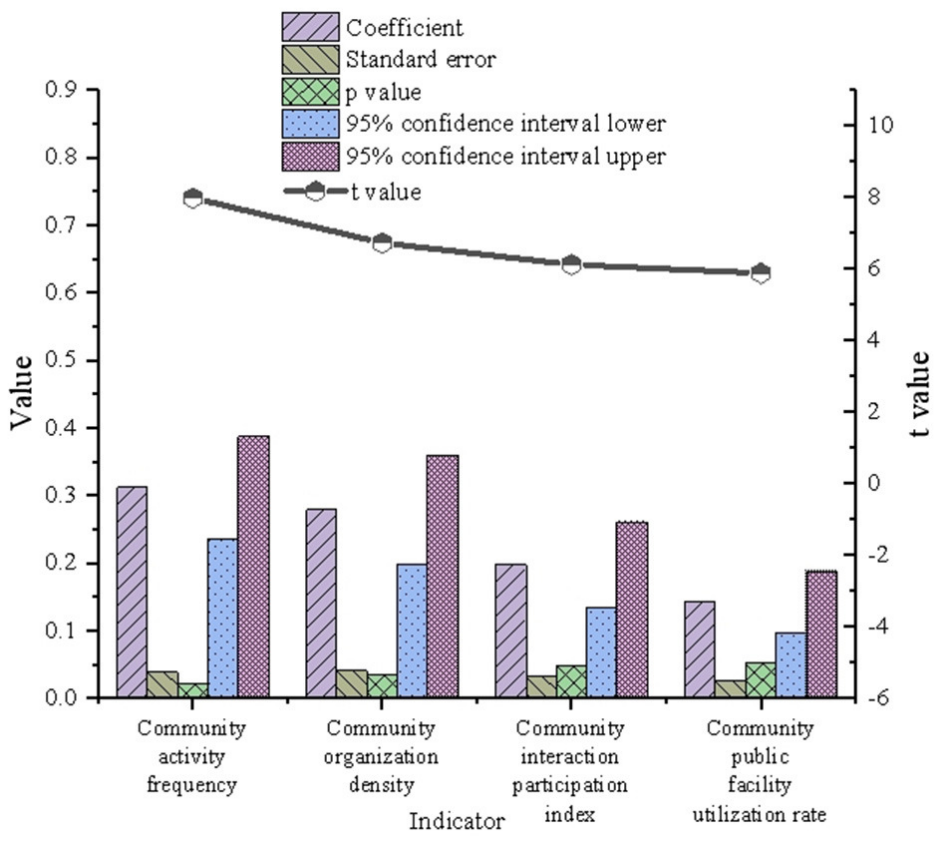
Verification results of multivariate model on community interaction in Wuhan.

The verification and analysis results of multivariate model on Wuhan community interaction are indicated in Figure 6.

In Figure 6, the regression coefficients of community activity frequency and community organization density are relatively high, at 0.312 and 0.279, respectively, indicating that these factors have a substantial influence on urban green space. Conversely, the community interaction participation index and the utilization of community public facilities exhibit lower coefficients of 0.198 and 0.143, respectively, suggesting a more limited impact. Additionally, analysis of the standard error and *t*-values reveals that community activity frequency and community organization density have low standard errors and high *t*-values, underscoring the reliability and statistical significance of their estimates. In contrast, the higher standard errors and lower *t*-values associated with the community interaction participation index and the utilization of public facilities indicate that their estimates are less reliable and statistically significant. Overall, the frequency of community activities and the density of community organizations emerge as key drivers of urban green space optimization, whereas the influence of community interaction participation and public facility utilization remains comparatively modest.

The findings further emphasize the significant positive impact of community activity frequency and community organization density, highlighting the pivotal role of green spaces within the community ecosystem. These spaces not only facilitate resident interactions and enhance social cohesion but also strengthen the social value of urban green spaces. A deeper analysis reveals that increased community activity frequency correlates with more effective utilization of green spaces. Regular cultural and sports events, as well as everyday neighborhood interactions, contribute to fostering a strong sense of community belonging and active engagement. In contrast, lower indices of community interaction and public facility utilization suggest limited social participation among residents in certain areas, potentially reflecting inequities in the distribution of public resources. This shortfall may diminish the role of green spaces in fostering community vitality and improving residents' quality of life. These findings highlight deficiencies in the multifunctionality and equity of green space planning, particularly concerning the alignment of resident needs with spatial design. To address these issues, strategies such as the equitable allocation of community public facilities, increased investment in activity-oriented green spaces, and the promotion of diverse community governance participation can enhance the multifunctional potential of green spaces. These measures would allow green spaces to contribute more effectively to community ecosystems, achieving synergistic development across social, ecological, and environmental dimensions.

### 5.4 Performance verification analysis of multivariable models

**Figure 7:**
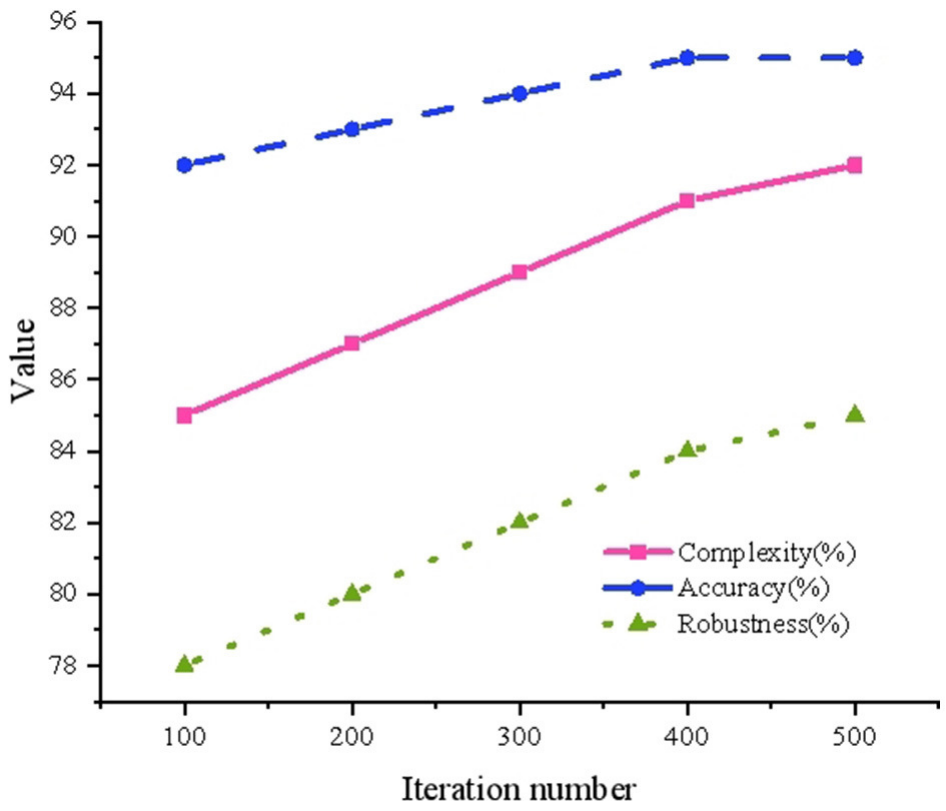
Performance verification analysis results of multivariate models.

The performance verification analysis results of multivariable models are suggested in Figure 7.

In Figure 7, an increase in the number of iterations corresponds to a gradual rise in model complexity, from 0.85 to 0.92, accompanied by a steady improvement in accuracy, which increases from 0.92 to 0.95. These trends indicate a progressive enhancement in the model's fitting capability and predictive performance. Simultaneously, the robustness index demonstrates an improvement from 0.78 to 0.85, suggesting increased model stability when applied to diverse datasets. These findings underscore that, even after multiple iterations, the multivariate model maintains high levels of accuracy and stability. Moreover, the model exhibits adaptability to complex data structures and variable environmental conditions, reinforcing its utility for dynamic prediction scenarios.

### 5.5 Analysis of the influence of the multivariate model on the economic benefit and social structure of Wuhan

**Figure 8:**
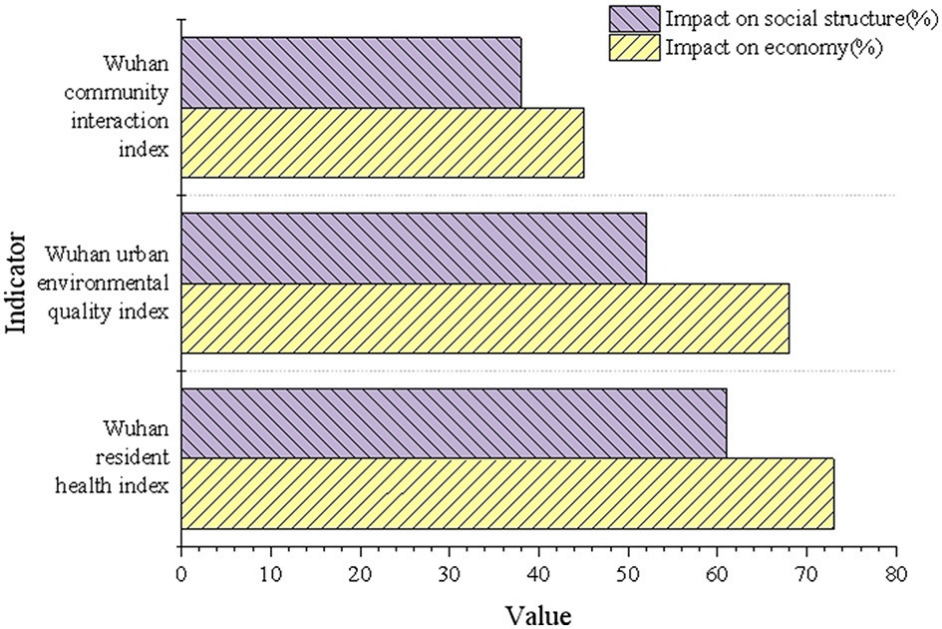
Analysis results of economic benefits and social structure impact.

The multivariate model's analysis results of the economic benefits and social structure of Wuhan are depicted in Figure 8.

In Figure 8, the optimization of health indices resulted in a 73% and 61% increase in the economic benefits and social structure impacts, respectively, within Wuhan's urban areas. Similarly, the environmental quality indices improved by 68% and 52%. These findings indicate that the optimization of green spaces significantly drives urban economic development and enhances social structures. Although the improvement rate of community interaction indices is relatively lower, its positive impact on economic and social structures reinforces the integral role of green spaces in the comprehensive enhancement of urban functions.

A deeper analysis reveals that green spaces mitigate urban heat island effects, directly reducing the adverse impacts of high temperatures on residents' productivity and daily lives. This contributes to a reduction in economic costs associated with energy consumption and medical expenses. Moreover, green spaces play a pivotal role in carbon sequestration by absorbing carbon dioxide, thereby providing crucial support for Wuhan's transition to a low-carbon economy. Additionally, rational green space layout and optimized distribution improve community interaction and social cohesion, making urban environments more adaptable to the dynamic demands of a climate-change context.

At the intersection of climate change adaptation and healthy city construction, the study demonstrates that integrating multidimensional dynamic optimization models can better align Wuhan's green space planning with ecological, economic, and social objectives. For high-density urban areas, priority should be given to the development of climate-adaptive ecological buffer zones. Enhancing vegetation diversity and improving green space accessibility can further maximize the synergistic effects of green spaces in improving resident wellbeing and promoting social stability. This optimization pathway not only strengthens the theoretical framework of the study but also provides robust support for future policy implementation.

## 6 Discussion

### 6.1 Research contributions

The findings of this study not only demonstrate the high applicability of the proposed model but also provide empirical data to better understand the multidimensional impacts of green spaces on urban health and environmental quality. These results hold significant reference value for similar urban contexts. Domestically, the study offers practical guidance for optimizing green space layouts in rapidly urbanizing, densely populated mid-to-large cities with limited green resources, such as Chongqing and Nanjing. These cities face considerable ecological pressures and an increasing demand for public health resources. By rationally allocating green spaces, improvements in quality of life and ecosystem service functions can be achieved to some extent. Internationally, for rapidly developing cities in Southeast Asia, such as Bangkok and Ho Chi Minh City, the study provides data-driven optimization pathways to address challenges such as uneven green space distribution, environmental pollution, and public health pressures. Additionally, for mid-sized cities with high population density, such as Budapest and São Paulo, as well as resource-constrained smaller cities, the machine learning techniques proposed in this study can precisely capture critical indicators to support scientific decision-making. This study deepens the understanding of healthy city metrics and offers universal solutions for green space management and planning across diverse global urban contexts. By doing so, it contributes to advancing sustainable urban development and improving urban resilience on a global scale.

The findings of this study are contextualized within the framework of existing research. The observed positive effects of health conditions and life expectancy closely align with the conclusions of Wu (7), who demonstrated that green spaces significantly enhance residents' quality of life by improving environmental quality and mental health. Similarly, the negative correlation between green space availability and chronic disease incidence supports the perspective that insufficient green spaces exacerbate exposure to pollution, thereby contributing to adverse health outcomes. Notably, the weaker influence of health awareness, a phenomenon seldom explored in depth in the literature, may be attributed to the underappreciation or limited utilization of green spaces by the public. This finding offers a promising avenue for future research to explore strategies for fostering greater awareness and engagement with green spaces. In terms of environmental quality, the results corroborate Tella and Balogun's (8) findings that noise pollution reduces the attractiveness and functionality of green spaces. Additionally, the significant negative impact of soil pollution on green space expansion, as identified in this study, underscores the critical need for ecological restoration strategies tailored to urban contexts. These findings provide a robust foundation for implementing targeted interventions, such as establishing ecological barriers to mitigate noise pollution and employing phytoremediation technologies to address soil contamination, thereby enhancing the sustainability and functionality of green spaces in Wuhan.

Considering these insights, and in light of Wuhan's specific urban dynamics, urban planners are encouraged to prioritize key indices identified in this study. For instance, leveraging the significant positive effects of health conditions and life expectancy aligns with the principles of the “healthy city” concept and can inform green space optimization strategies. In districts with limited green resources, the development of community parks and health trails can directly address residents' health needs and promote equitable access to green spaces. Furthermore, the strong correlations between air and water quality indices and environmental quality underscore the potential of green spaces to mitigate pollution. Urban planners can integrate these findings into the design of ecological barriers or vegetative buffers in areas with high pollution levels to enhance the regulatory functions of green spaces. Simultaneously, coordinated efforts in pollution control and green space restoration initiatives should be promoted to achieve synergistic benefits for the environment and public health.

When analyzed through the lens of academic advancements, this study significantly contributes to the exploration of the multifaceted functions of green spaces in the context of climate change. For instance, Pinto et al. (47) highlighted the link between urban heat island effects and extreme heat events, emphasizing the necessity of nature-based solutions to mitigate these phenomena. This study aligned closely with their perspective by demonstrating how optimized green space layouts effectively reduced urban heat island effects, providing region-specific insights for climate-adaptive urban planning. Similarly, Cherif et al. (48), through meta-analyses, revealed the temperature-regulating roles of green spaces under varying climatic conditions. Building on this, the findings of this study validated the substantial positive impact of green spaces in mitigating environmental stressors such as high pollution and noise levels, underscoring the regional specificity of these benefits. Furthermore, Han et al. (49), employing spatial econometric analyses, established that green spaces alleviated pollutants such as PM2.5. This study expands on their work by emphasizing the synergistic role of green ecosystems in simultaneously improving environmental quality and enhancing residents' wellbeing, thereby enriching the theoretical framework of green space ecosystem services. In summary, this study not only addresses a critical gap in the intersection of urban health and climate change research but also provides multidimensional data-driven insights to support policy formulation. It exemplifies the integration of academic and practical value, advancing the application of green spaces in building urban climate resilience and offering actionable strategies for green space management in diverse global urban contexts.

### 6.2 Limitations and future directions

Nevertheless, it is important to acknowledge certain limitations in this study that may affect the generalizability of the results. Firstly, the reliance on publicly available datasets introduces potential biases in data completeness and quality. For instance, the uneven spatial distribution of data points may lead to the underrepresentation or omission of certain areas, thereby weakening the model's performance in ensuring regional balance. Furthermore, the selection of health indicators was limited to quantifiable data, and non-quantifiable dimensions such as subjective wellbeing and mental health were not incorporated, which may constrain the comprehensive understanding of the social benefits of green spaces. Secondly, the assumption of linear relationships between variables oversimplifies the complexity of real-world interactions. The effects of green spaces on multidimensional indicators often exhibit non-linear characteristics; for example, health indicators may show non-linear jumps under certain threshold conditions, which linear analytical methods may fail to capture comprehensively. Lastly, the study's temporal and spatial scope was somewhat fixed, failing to account for the dynamic trends of green space usage across different seasons or over long-term urban planning periods. This raises the need for further research to evaluate the long-term effects of green spaces more comprehensively.

To overcome these limitations, future research should explore deeper advancements in data sources, methodological innovations, and the expansion of analytical scope. First, real-time data monitoring technologies should be integrated to enhance the dynamic nature and precision of the data, alongside community-based participatory approaches for data collection. This would ensure the inclusion of residents' subjective perceptions and cultural aspects, capturing the social benefits of green spaces and their adaptability to diverse population needs. Second, in terms of methodological innovation, future studies should integrate deep learning models or hybrid methods, applying non-linear analysis techniques to explore the relationships between multidimensional variables, thus providing a more comprehensive reflection of complex dynamic interactions. Such techniques can identify potential non-linear associations and multi-level mechanisms between variables, offering stronger support for the accuracy and interpretability of predictive outcomes. Furthermore, expanding the temporal and spatial scope of analysis is a critical direction for future development. For example, long-term data analysis could capture the dynamic trends of green space across different seasons and development stages, further revealing its potential contribution to addressing climate change and optimizing urban wellbeing (50). In summary, future research should drive the integration of data, methods, and scope optimization, fostering a deeper convergence between theory and practice in green space studies, and providing a more comprehensive scientific foundation for sustainable urban development.

## 7 Conclusion

This study explores the complex relationship between the optimization of urban green spaces in Wuhan and the multidimensional indices of a healthy city through the application of a multivariate model. By integrating RF with SVM algorithms, this study makes significant strides in quantifying the role of key indices and uncovering their interaction mechanisms. The findings demonstrate that green spaces are essential in improving residents' health and extending life expectancy. Additionally, these spaces play a crucial role in enhancing urban environmental quality and fostering community interaction. This study not only enriches the theoretical understanding of the interactive mechanisms between green spaces and healthy city development, but also provides critical empirical evidence to guide green space and urban planning efforts in Wuhan. Through a systematic analysis of indices such as environmental quality, community interaction, and health conditions, the study emphasizes the irreplaceable role of green spaces as a central element of urban resilience and social wellbeing.

From a practical perspective, the findings offer valuable decision-making support for urban planning in Wuhan within the framework of a healthy city. Positive indices, such as health conditions and life expectancy, highlight the potential of green spaces to optimize the living environment and underscore their importance in promoting ecosystem sustainability and improving residents' wellbeing. Furthermore, this study outlines specific strategies for addressing negative impacts such as noise and soil pollution, recommending the construction of ecological barriers and the promotion of phytoremediation technologies. These strategies provide practical pathways for balancing green space development with urban growth objectives. Additionally, the model architecture and empirical methods utilized in this study demonstrate strong generalizability, offering a reference framework and practical guidance for other cities facing similar challenges in green space optimization. Moving forward, the development and optimization of green spaces should not only focus on enhancing ecological and health benefits, but also on integrating them more deeply into social and cultural dimensions. This approach aims to fully harness the multifaceted contributions of green spaces to urban health, ecological balance, and social development.

## Data Availability

The original contributions presented in the study are included in the article/supplementary material, further inquiries can be directed to the corresponding author.
